# Understanding the Impact of Sheep Digestion on Seed Germination in the Cold Desert Annual *Diptychocarpus strictus* with Emphasis on Fruit and Seed Heteromorphism

**DOI:** 10.3390/life14020235

**Published:** 2024-02-08

**Authors:** Xuheng Zhao, Yixiang Wang, Fangfang Cao, Xuzhe Wang, Fanfan Zhang, Sumera Anwar, Zhihua Sun, Hongsu He

**Affiliations:** 1 College of Animal Science and Technology, Shihezi University, Shihezi 832000, China; 15517920326@163.com (X.Z.); wyx18369499053@163.com (Y.W.); 18899598816@163.com (F.C.); zhangfanfan@shzu.edu.cn (F.Z.); 2 Department of Botany, Government College Women University, Faisalabad 38000, Pakistan; sumeraanwar@mail.hzau.edu.cn

**Keywords:** *Diptychocarpus strictus*, heteromorphism, simulation of digestion, germination behavior

## Abstract

This study aimed to investigate the morphological characteristics of fruits and seeds from *Diptychocarpus strictus*, a plant species inhabiting the cold desert pastoral area of China. Furthermore, this study sought to evaluate the germination potential of these seeds following digestion by sheep. This study employed the sheep rumen fistula method to simulate rumen digestion at various time intervals. Subsequently, an in vitro simulation method was utilized to simulate true gastric and intestinal digestion after rumen digestion. Paper germination tests were then conducted to assess the impact of the digestive process on the heteromorphic seed morphology and germination. During rumen digestion, the seeds were protected by wide wings. The results revealed a highly significant negative correlation (*p* < 0.01) between seed wing length and digestion time. Post-rumen digestion, variations in the germination rate among seeds from fruits at different locations were observed. Indicators, such as germination rate, exhibited a highly significant negative correlation with rumen digestion time (*p* < 0.01). In vitro simulated digestion tests demonstrated that *Diptychocarpus strictus* seeds retained their ability to germinate even after complete digestion within the livestock’s digestive tract. The polymorphic nature of *Diptychocarpus strictus* seeds, coupled with their capacity to survive and germinate through the digestive tract, facilitates the spread of these seeds. This finding has implications for mitigating desert grassland degradation and promoting sustainable ecological development.

## 1. Introduction

Desert grasslands play a pivotal role in terrestrial ecosystems, serving as critical indicators of grassland desertification and ecological environment changes. Moreover, they provide essential grazing pastures for livestock during spring and autumn, experiencing substantial grazing pressure. Unfortunately, unsustainable usage of desert grassland in recent years has led to severe degradation. Consequently, research focusing on the dynamics of change and restoration of desert grassland due to grazing has become a prominent area of concern [1].

In grazing ecosystems, interactions between grasses and animals are of paramount importance. These interactions involve animals foraging for seeds, which are then transported through their digestive tracts and subsequently excreted, leading to extensive seed displacement in space. Livestock, in particular, act as crucial agents in selecting suitable germination sites for grassland plants, aiding successful seedling establishment, and contributing to the development of new habitats [2,3]. The appealing taste of fruits attracts various fruit-eating animals, such as birds [4], primates [5], and rodents [6], facilitating seed dispersal by animals. Therefore, early research on seed dispersal through the digestive tracts of animals focused on fruit-eating animals. As research has progressed, attention has now shifted towards understanding how seeds without pulpy coverings are dispersed through the digestive tracts in grassland ecosystems.

For successful seed dispersal through the digestive tracts of livestock, it is crucial that the seeds have the ability to germinate after digestion, and their viability can be influenced by morphological differences [7,8]. In desert pastoral areas with a long history of livestock, plant species are adapted to grazing, enhancing seed dispersal, increasing species diversity, and promoting spatial uniformity. Therefore, seeds have a special role in colonization processes and regeneration [9]. Seed heteromorphism, also known as heterotypy, is a unique plant adaptation to the ecological environment. It plays a crucial role in studying ecological adaptation mechanisms, life-history responses, and plant evolution [10,11]. *Diptychocarpus strictus*, a polymorphic plant belonging to the Brassicaceae family, is a common high-quality grass of the Junggar desert pastoral area in the spring and autumn. Previous studies have explored its polymorphism, reproductive allocation, and ecological adaptation [12,13,14], as well as seed dispersal, dormancy, and germination in natural grasslands [15,16,17]. In this study, we chose *Diptychocarpus strictus* to study the grass–livestock relationship. Our goal was to investigate how seed heteromorphism enables the plant to withstand the effects of grass–livestock interactions in grazing ecosystems. Additionally, we aimed to determine whether the seeds can still germinate after being consumed and digested by livestock. These findings have implications for renewing plant populations and mitigating desert grassland degradation.

## 2. Materials and Methods

### 2.1. Experimental Site

This study was conducted in the temperate desert pastoral region of the Ziniquan sheep breeding farm, situated on the north slope of the Tianshan Mountains (43°26′–45°20′ N, 84°58′–86°24′ E) in Shihezi. The area, experiencing an annual precipitation of 150–200 mm and a mean annual temperature ranging from 8–10 °C, is characterized by extremely high temperatures that can surpass 40 °C. The soil in this region is identified as typical light chestnut-covered soil with a loess parent material and a deep soil layer. The dominant vegetation in the study area is *Seriphidium transiliense*, accompanied by ephemeral annuals and quasi-ephemeral perennials. The climate mirrors that of Kazakhstan in Central Asia, and the region is a crucial grazing area during spring and autumn, subject to substantial grazing pressure. Unfortunately, degradation has been experienced in recent years primarily due to the deterioration of the climatic environment [18,19].

### 2.2. Plant Morphology of Diptychocarpus strictus 

**Figure 1:**
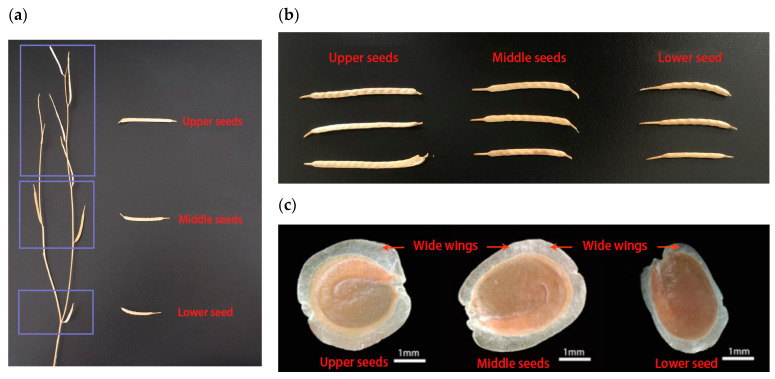
(**a**) Morphology of the upper, middle, and lower silique on the plant, (**b**) silique morphology, and (**c**) seed morphology of *Diptychocarpus strictus*.

Mature, intact fruits (including seeds and accessory structures) were collected from *Diptychocarpus strictus* (Fisch. ex M.Bieb.) Trautv. plants in the study area between late April and early June 2022. Following a predetermined protocol (refer to Figure 1), the upper, middle, and lower fruits were individually collected, naturally dried, sorted, and stored in sealed bags for subsequent trials. The upper fruit is dorsoventrally compressed and dehisces immediately after ripening. The middle fruit, comparatively shorter than the upper fruit, is also dorsoventrally compressed and dehisces upon ripening. In contrast, the lower fruit is subterete, full, and short and becomes highly lignified when ripe and lacking dehiscence.

All three forms of fruit exhibit an earthy yellow color, featuring a distinct beak and stalk. The seeds within these fruits are brown and possess a compressed ellipsoid or ovoid shape. Notably, transparent seed wings are present on the edges of the seeds adding distinct morphology of *Diptychocarpus strictus*.

### 2.3. Sampling and Measurements

To assess the impact of different treatments on heterocarpic *Diptychocarpus strictus* seeds, various conditions were applied based on the natural environment and the feeding patterns of livestock. The treatments included dehiscent upper fruit (DU), artificially crushed upper fruit (simulating livestock feeding, the description given below) (ACU), dehiscent middle fruit (DM), artificially crushed middle fruit (ACM), un-dehisced lower fruit (UDL), and artificially crushed lower fruit (ACL).

### 2.4. Simulated Digestion Test

The simulated digestion test consisted of two parts. The first part involved simulating rumen digestion using the rumen fistula nylon bag method [19]. The second part simulated digestion from the wrinkled stomach to the large intestine using an in vitro simulation [20,21]. Six treatment times were established to simulate rumen digestion, 6, 12, 24, 36, 48, and 72 h, with the control group (CK) representing 0 h. After each treatment time, the seeds were extracted from the rumen of three sheep as replicates, rinsed with tap water, naturally dried, and stored in sealed bags. Morphological differences were then observed in the seeds at different digestion times. 

A portion of the seeds underwent post-digestion somatic microscopic observation and germination tests. The remaining seeds underwent in vitro simulated digestion from the wrinkled stomach to the large intestine. This involved preparing a 2 L solution in a beaker containing 3.3 g of pepsin (1:3000), and pH was adjusted to 2.5 with 0.1 N HCL and mixed evenly. The seeds, treated with different digestion times in the rumen, were placed in the beaker and then shaken at 39 °C for 2 h to simulate sheep abomasum digestion. After this, the pH of the solution was adjusted to 7.5 using KH_2_PO_4_ buffer, and 2.4 g of trypsin (1:250) was added. After further shaking at 39 °C for 2 h to simulate sheep small intestine digestion, the seeds were placed in a solution composed of 1/5 fresh sheep feces (obtained from fistula sheep) and 4/5 phosphate buffer (1:4:20 NH_4_CL:NaH_2_PO_4_·H_2_O:Na_2_HPO_4_). They were then subjected to a constant temperature shaker at 39 °C for 24 h to simulate sheep large intestine digestion. After thoroughly rinsing and air-drying, the seeds were ready for use. The control group only underwent in vitro simulated digestion. No significant difference in seed morphology was observed following in vitro simulated digestion and only seed germination experiments were conducted.

### 2.5. Germination Test

The seeds from various treatments were placed in 9 cm Petri dishes with double-layered filter paper in an intelligent artificial climate chamber (model: RXZ-1608). The conditions were set at 50% humidity, mimicking the climate of the *Diptychocarpus strictus* habitat, with a variable temperature cycle of 16 h of full light at 25 °C during the day and 8 h of darkness at 15 °C at night. Water was added at suitable intervals to maintain moisture, and germinating seeds (with a 2 mm radicle as the germination criterion) were recorded every 24 h for 14 days.

Ungerminated seeds underwent staining with tetrazolium to determine viability. Seeds were cut crosswise with a scalpel, soaked in a tetrazolium solution, and incubated at 30 °C for 30 min. Staining was observed and seeds with stained embryos were considered viable, while unstained ones were considered nonviable. Calculations for corresponding indicators were performed according to the following established formulas [22,23,24]:

$$Germination\;rate\left(\%\right)=\frac{Number\;of\;all\;germinated\;seeds\;at\;14\text{-}day}{Total\;number\;of\;seeds}\;\times \;100$$

$$Viability\;of\;ungerminated\;seeds\;(\%)\;=\;\frac{Number\;of\;stained\;seeds}{Number\;of\;ungerminated\;seeds}\;\times \;100$$

$$Germination\;index\;(GI)\;=\;\sum (\frac{Gt}{Dt})$$

$$Vitality\;index\;\left(VI\right)\;=\;GI\;\times \;S$$

where *Gt* is the number of germinations on day *t*; *Dt* is the number of days to germination; and *S* is the sum of the length of the germ and radicle at day 10.

### 2.6. Statistical Analysis

Data collected from the experiment were processed using Microsoft Excel (2021) and analyzed using SPSS 20.0. One-way ANOVA was utilized for statistical significance and multiple comparisons were conducted using Duncan’s method. Results were expressed as mean ± standard error, with *p* < 0.05 indicating significant differences. Pearson correlation analysis was conducted to evaluate the correlation between digestion time and seed germination rate, with a significance level set at *p* < 0.05 for significant differences and *p* < 0.01 for highly significant differences.

## 3. Results

### 3.1. Seed Morphology after Simulated Rumen Digestion

**Figure 2:**
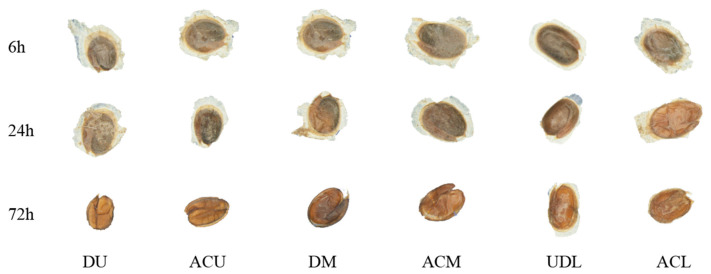
Seed morphology of *Diptychocarpus strictus* at different digestion times of simulated rumen digestion; DU: dehiscent upper fruit; ACU: artificially crushed upper fruit, DM: dehiscent middle fruit; ACM: artificially crushed middle fruit; UDL: un-dehisced lower fruit; ACL: artificially crushed lower fruit.

Seeds collected at different time intervals (6 h, 24 h, and 72 h) during simulated rumen digestion were examined using a stereomicroscope at 2× magnification (Figure 2). Seeds started losing mucilage after 6 h of digestion compared to the control at the start of digestion (Figure 1c). The observations revealed a progressive increase in digestion-related changes in the seed wings of seed appendages over time. After 72 h of digestion, only the un-dehiscent lower fruit (UDL) seeds retained some seed wings. 

### 3.2. Seed Germination Rate after Simulated Rumen Digestion

**Figure 3:**
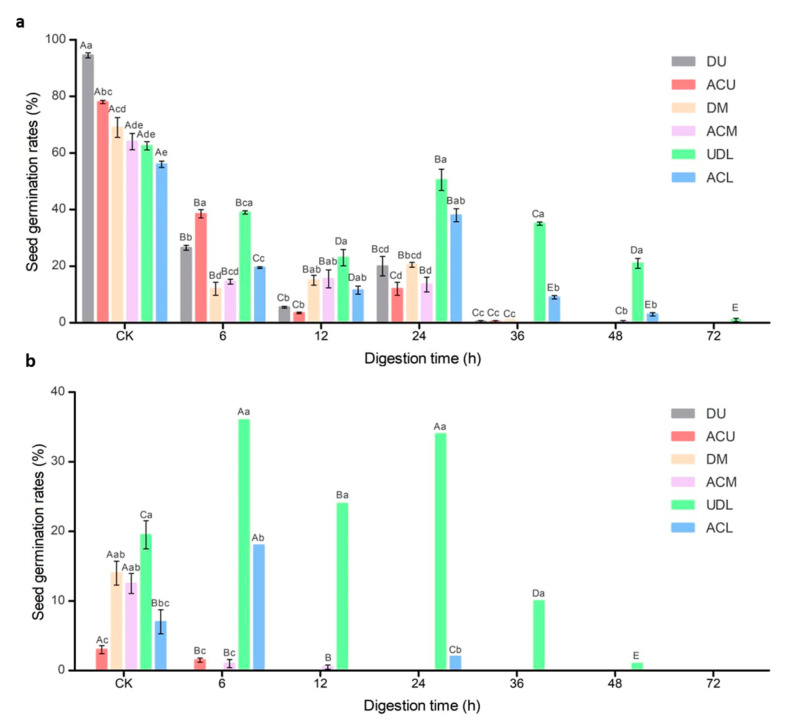
Seed germination rates at different digestion times of (**a**) rumen digestion and (**b**) in vitro mock digestion. Different capital letters indicate significant differences (*p* < 0.05) among hours, and different lowercase letters indicate significant differences (*p* < 0.05), among treatments. DU: dehiscent upper fruit; ACU: artificially crushed upper fruit, DM: dehiscent middle fruit; ACM: artificially crushed middle fruit; UDL: un-dehisced lower fruit; ACL: artificially crushed lower fruit.

The data presented in Figure 3a clearly illustrate that the germination rate of seeds taken from the upper portion of the plant was higher compared to the germination rate of seeds from the middle and lower parts of the fruit. As the duration of rumen digestion increased, an overall decline in the germination rate of seeds from different parts was evident. In particular, the germination rate of seeds from the upper and middle fruits significantly decreased at 36 h, 48 h, and 72 h of rumen digestion. Conversely, the germination rate of seeds from the lower fruit was higher than that of the upper and middle fruit seeds.

Furthermore, it was observed that the germination rate of artificially crushed seeds before rumen digestion was lower compared to seeds under natural conditions. Notably, the un-dehised lower fruit consistently displayed higher germination rates compared to the artificially crushed lower fruit. Additionally, the germination rates of seeds from un-dehised lower fruit increased after 24 h of rumen digestion.

### 3.3. Seed Germination Rate after In Vitro Mock Digestion

According to the data presented in Figure 3b, a significant reduction in the germination rates of seeds was observed under two distinct conditions: when exposed to in vitro mock digestion alone and when subjected to in vitro mock digestion after rumen digestion. In particular, seeds from the upper fruit after in vitro mock digestion did not germinate. Similarly, seeds from the upper and middle fruit that were subjected to rumen mock digestion for 24–72 h failed to germinate after subsequent in vitro mock digestion. Additionally, all seeds that underwent rumen mock digestion for 72 h also did not germinate. While the seeds of un-dehisced lower fruits exhibited better germination rates after in vitro simulated digestion.

### 3.4. Seed Viability of Ungerminated Seeds after Simulated Rumen Digestion

**Figure 4:**
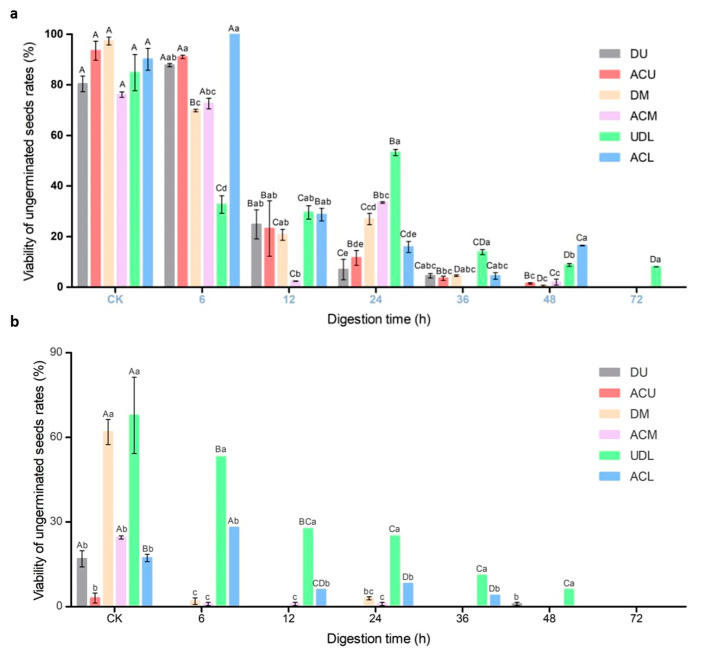
Viability of ungerminated seeds at different digestion times of (**a**) rumen digestion and (**b**) in vitro mock digestion. Different capital letters indicate significant differences (*p* < 0.05) among hours, and different lowercase letters indicate significant differences (*p* < 0.05), among treatments. DU: dehiscent upper fruit; ACU: artificially crushed upper fruit, DM: dehiscent middle fruit; ACM: artificially crushed middle fruit; UDL: un-dehisced lower fruit; ACL: artificially crushed lower fruit.

The data presented in Figure 4a reveal a consistent decline in the viability of ungerminated seeds across different parts as the duration of rumen digestion progresses. Initially, after 6 h of rumen digestion, the ungerminated seeds (excluding the un-dehisced lower fruit seeds) exhibited high viability. Even after 48 h of rumen digestion, the ungerminated seeds remained viable, except for the dehiscent upper fruit seeds. However, after 72 h of rumen digestion, only the un-dehisced lower fruit seeds retained their viability.

### 3.5. Seed Viability of Ungerminated Seeds after In Vitro Simulated Digestion

Figure 4b illustrates a consistent decline in the viability of ungerminated seeds. Notably, the un-dehisced seeds of the upper fruit maintained their vitality even after simulated digestion in vitro. Similarly, after 24 h of rumen digestion, the seeds of the middle fruit still exhibited viability. However, after 72 h of rumen digestion, the viability of germinated seeds in different parts of the rumen was no longer sustained.

### 3.6. Seed Germination Index after Simulated Rumen Digestion

**Figure 5:**
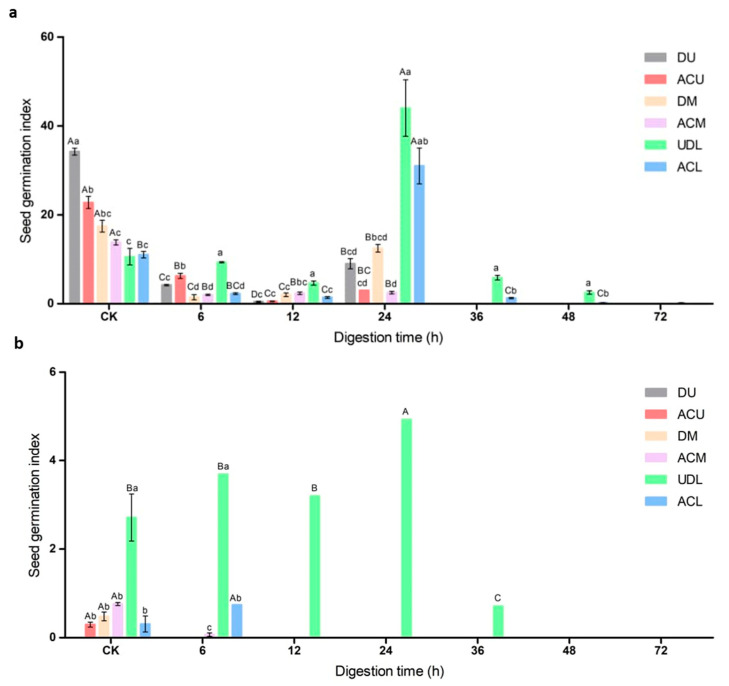
Seed germination index after different digestion times of (**a**) rumen digestion and (**b**) in vitro mock digestion. Different capital letters indicate significant differences (*p* < 0.05) among hours, and different lowercase letters indicate significant differences (*p* < 0.05), among treatments. DU: dehiscent upper fruit; ACU: artificially crushed upper fruit, DM: dehiscent middle fruit; ACM: artificially crushed middle fruit; UDL: un-dehisced lower fruit; ACL: artificially crushed lower fruit.

As shown in Figure 5a, the germination indices of *Diptychocarpus strictus* seeds showed a decreasing trend with increasing rumen digestion time, with a brief peak increase after 24 h of rumen digestion. There was no germination index for the upper and middle fruit seeds after 36–72 h of rumen digestion. The germination index of the undigested upper fruit seeds was higher than that of the middle and lower fruit. The germination index of the lower fruit seeds was significantly higher than that of the upper and middle fruit seeds after 24–72 h of rumen digestion.

### 3.7. Seed Germination Index under In Vitro Simulated Digestion

The in vitro simulated digestion caused a significant reduction in the germination index of *Diptychocarpus strictus* seeds (Figure 5b). There was no germination index for dehiscent upper fruit seeds before and after in vitro digestion. The rumen-digested upper and middle fruit seeds showed no germination index after in vitro simulated digestion, excluding artificially crushed middle fruit seeds digested for 6 h in the rumen. After rumen digestion for 12 to 72 h, there was no germination index for the artificially crushed lower fruit seeds. After 48 and 72 h of rumen digestion, different parts of the seeds did not have a germination index.

### 3.8. Seed Vitality Index after Simulated Rumen Digestion

**Figure 6:**
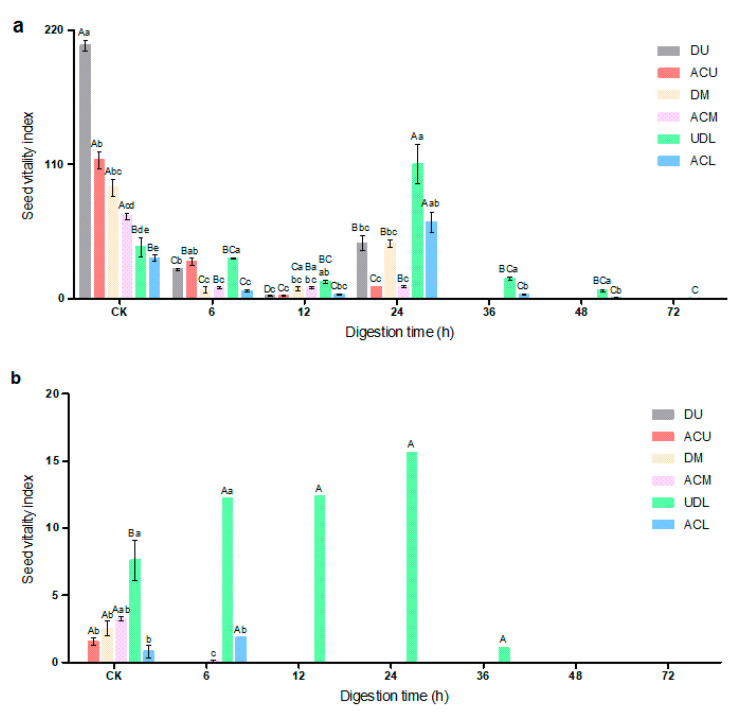
Seed vitality index after different digestion times of (**a**) rumen digestion and (**b**) in vitro mock digestion. Different capital letters indicate significant differences (*p* < 0.05) among hours, and different lowercase letters indicate significant differences (*p* < 0.05), among treatments. DU: dehiscent upper fruit; ACU: artificially crushed upper fruit, DM: dehiscent middle fruit; ACM: artificially crushed middle fruit; UDL: un-dehisced lower fruit; ACL: artificially crushed lower fruit.

The vitality index of the seeds of *Diptychocarpus strictus* was reduced after rumen digestion, and further reduction was observed as time progressed (Figure 6a). There was no vitality index for upper and middle fruit seeds digested by the rumen for 36–72 h. The undigested upper fruit seeds showed the best vitality index. After 24 h of rumen digestion, the vitality index of lower fruit seeds was the highest.

### 3.9. Seed Vitality Index after In Vitro Simulated Digestion

The vitality index of *Diptychocarpus strictus* seeds decreased substantially after in vitro simulated digestion (Figure 6b). The un-dehisced lower fruit seeds subjected to rumen digestion and in vitro mock digestion had a better vitality index compared to the other treatments. The dehiscent upper fruit seeds that were digested in vitro had no vitality. The upper fruit, middle fruit, and artificially crushed lower fruit seeds digested in the rumen for 12–72 h had no vitality index after simulated digestion in vitro. There was no vitality index in different parts of the seeds after 48 and 72 h of rumen digestion.

### 3.10. Correlation Analysis

**Figure 7:**
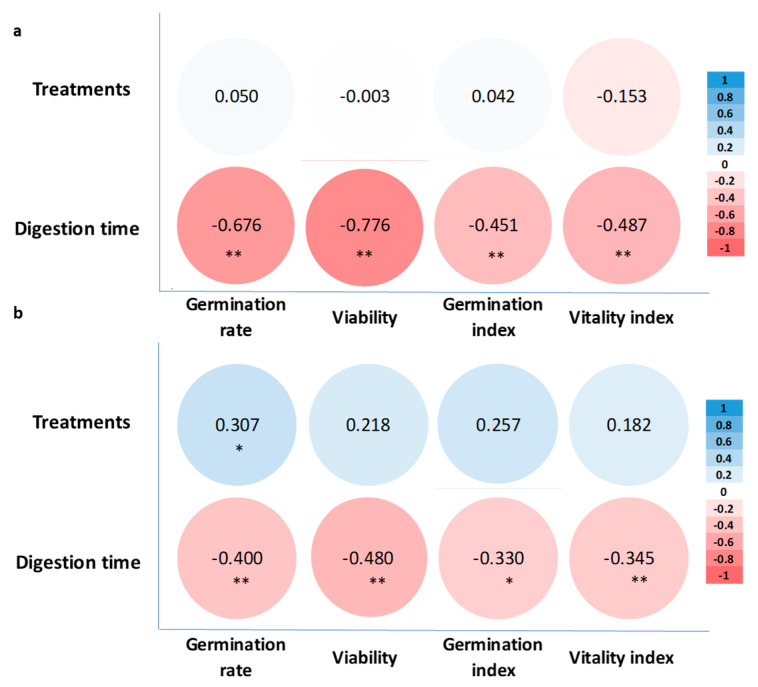
Correlation of seed germination indicators with different digestion times of (**a**) rumen digestion and (**b**) in vitro digestion. * indicates a significant correlation at the level of *p* < 0.05; ** indicates a significant correlation at the level of *p* < 0.01.

According to Figure 7a, there was no significant correlation between treatment and seed germination traits for rumen digestion. However, a highly significant negative correlation was observed between germination rate, viability of ungerminated seeds, germination index, vitality index, and rumen digestion time (*p* < 0.01). 

Figure 7b reveals a significant positive correlation between the seed treatment and germination rate (*p* < 0.05) for in vitro digestion. Additionally, a highly significant negative correlation was observed between germination rate, viability of ungerminated seeds, vitality index, and rumen digestion time (*p* < 0.01), while a significant negative correlation was observed between germination index and rumen digestion time (*p* < 0.05). 

## 4. Discussion

### 4.1. Changes in Seed Morphology after Simulated Rumen Digestion

Seed heterogeneity can manifest in variations in size, quality, shape, color, or dispersal structure and can impact dormancy, dispersal ability, seed bank longevity, viability during storage, and germination behavior. Some of this diversity is associated with fruit morphology and the level of armament, as evidenced by studies on Australian aubergines. In these studies, the presence of burrs on the fruit facilitated seed dispersal [25,26]. The reason why rumen digestion did not affect the interior of the heteromorphic seeds of *Diptychocarpus strictus* is due to the presence of the seed wing. 

During rumen digestion, the seed wings protect the seeds, and as digestion time increases, the seed wings gradually undergo digestion while the main seed structures are preserved, which is necessary for population dispersal. However, there is a trade-off involved. The seed wings have been previously documented to possess the ability to rapidly absorb water. They form mucilage structures within approximately 40 min, retaining water for up to 5 h. The formation of these mucilage structures serves dual purposes, storing water for the seeds and providing a protective layer, thereby creating favorable conditions for seed germination and establishment. This information aligns with findings from prior studies [27]. Consequently, the digestion of seed wings by the rumen may also have a negative impact on seed germination and establishment.

### 4.2. Seed Germination Behavior after Simulated Digestion

Differences in seed morphology can influence germination patterns [9]. For instance, the germination rate of dehiscent upper fruits decreased as digestion time increased. However, after 24 h of digestion, the germination rate increased due to the disruption of the seed wing structure, which exposed the seeds directly to germination conditions. No germination occurred after 48 h of digestion, despite the internal structure of the seeds remaining intact. This lack of germination can be attributed to the inhibitory effect of rumen fluid on the seeds [28].

The artificially crushed upper fruit seeds were used to simulate the feeding process of livestock. These seeds experienced disruption during rubbing, resulting in a lower germination rate compared to the dehiscent upper fruit seeds. Correlation analysis indicated that the treatment method had a minimal impact on seed production, while the degree of damage caused to the seeds by different digestion times had the greatest influence on seed germination.

During rumen digestion, the morphological structure of the middle fruit closely resembles that of the upper fruit, and their germination behavior does not significantly differ. On the other hand, the lower fruits are challenging to dehisce and possess more lignified structures. Consequently, the un-dehisced lower fruit seeds continue to germinate even after 72 h of digestion. Among the polymorphic seeds, each type plays a distinct role. The presence of the lower fruit in *Diptychocarpus strictus* serves as a safeguard for the population’s seed bank, as it cannot disperse independently when mature. The digestive tract of animals and the structural characteristics of the lower fruit enable the long-distance dispersal of *Diptychocarpus strictus*.

The Initial step for the successful dispersal of seeds within animals involves the survival of seeds throughout various digestive processes, including chewing, regurgitation, and digestion. Intestinal digestion can also impact seed dormancy, potentially influencing germination success and duration. Animal excreta contains substances that inhibit growth, such as phenolic compounds and fatty acids. These phytotoxic compounds have the potential to alter the activity of germination-regulating enzymes, thereby inhibiting germination in certain plant species [29].

Through the ruminal digestion of seeds, followed by in vitro simulated digestion, it was observed that heterocarpic mustard seeds, regardless of the treatment they received, retained their ability to germinate even after undergoing the wrinkled stomach and intestinal digestion processes. Although the dehiscent upper fruits lost their germination ability, their ungerminated seeds remained viable, indicating that the in vitro digestion did not fully destroy the seeds’ viability. The relationship between the germination rate and digestion time was further confirmed through in vitro simulated digestion. The viability of ungerminated seeds, germination index, and vitality index all supported the germination rate, providing additional insights into the effects of digestion on the seeds [30,31].

## 5. Conclusions

In conclusion, the successful germination of *Diptychocarpus strictus* seeds following simulated digestion tests suggests that these seeds possess effective protection from both external and internal structures. This resilience enables them to withstand consumption by sheep, irrespective of whether the fruit is dehiscent or not. This finding confirms that *Diptychocarpus strictus* seeds can effectively disperse through animal consumption in natural environments. The implications of this research are significant for studying methods to mitigate desert grassland degradation and restore desert vegetation.

## Data Availability

The presented data are available on request from the corresponding author.
